# Neutral and Non-Neutral Evolution of Duplicated Genes with Gene Conversion

**DOI:** 10.3390/genes2010191

**Published:** 2011-02-18

**Authors:** Jeffrey A. Fawcett, Hideki Innan

**Affiliations:** 1 Graduate University for Advanced Studies, Hayama, Kanagawa 240-0193, Japan;E-Mail: fawcett@soken.ac.jp; 2 PRESTO, Japan Science and Technology Agency, Saitama 332-0012, Japan

**Keywords:** gene duplication, gene conversion, neutral, selection, mutation, evolution, theory

## Abstract

Gene conversion is one of the major mutational mechanisms involved in the DNA sequence evolution of duplicated genes. It contributes to create unique patters of DNA polymorphism within species and divergence between species. A typical pattern is so-called concerted evolution, in which the divergence between duplicates is maintained low for a long time because of frequent exchanges of DNA fragments. In addition, gene conversion affects the DNA evolution of duplicates in various ways especially when selection operates. Here, we review theoretical models to understand the evolution of duplicates in both neutral and non-neutral cases. We also explain how these theories contribute to interpreting real polymorphism and divergence data by using some intriguing examples.

## 1. Introduction

Our understanding of molecular evolution requires a good understanding of the evolutionary outcome of various mutational mechanisms under neutrality and under the pressure of selection. One well-studied mutational mechanism that appears to have largely contributed to the evolution of most organisms is gene duplication [1–5]. However, when studying the evolution of duplicated genes, the assumption that homologous sequences evolve independently does not always hold, despite being a frequent assumption in molecular evolutionary studies. This is because of a process called gene conversion, which allows the co-evolution of duplicated genes (*i.e.*, concerted evolution). Several studies have suggested that gene conversion might have a non-negligible effect on the evolution of duplicated genes [6–13]. Thus, our understanding of the evolutionary significance of gene duplication will not be complete without considering the contribution of gene conversion [14,15].

Gene conversion is a non-reciprocal recombination process, commonly described as a “copy-and-paste” event. In contrast to recombination which results in the sequences of the two strands being exchanged, gene conversion results in one sequence replacing the other. As a consequence, the two sequences become identical (see the reviews by [16] and [17] in this issue). Gene conversion can occur between alleles (intralocus or allelic gene conversion) instead of recombination, but can also occur between duplicated sequences (paralogs) that share high similarity. Such interlocus (or nonallelic, ectopic) gene conversion helps to create unique patterns of evolution of the DNA sequences of duplicates, most notably the pattern of sequence divergence and polymorphism.

Here, we first review how gene conversion affects the molecular evolution of duplicates under neutrality. Then, we describe some positive and negative effects of gene conversion, and discuss the joint effect of selection and gene conversion on the evolution of duplicated genes. We also consider how DNA sequence data should be interpreted by using several examples.

## 2. Neutral Evolution of Duplicates with Gene Conversion

### 2.1. Gene Conversion between Paralogs

**Figure 1:**
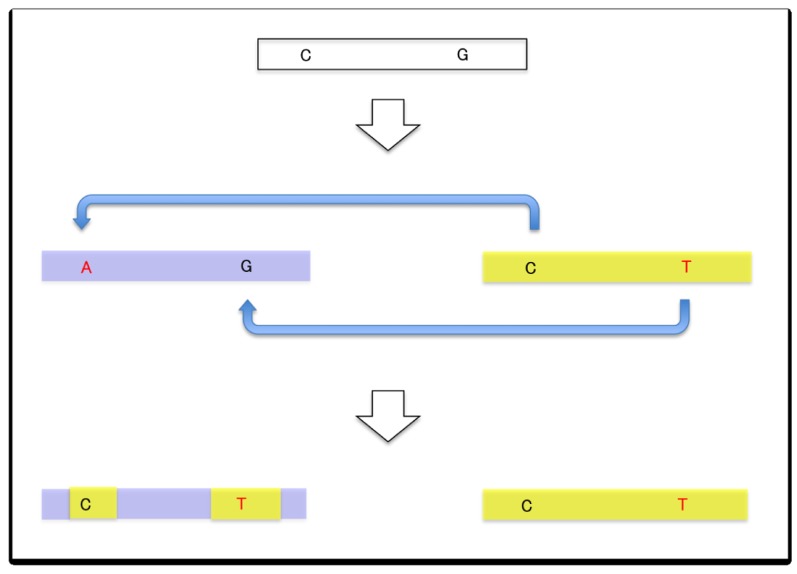
Simple gene conversion model for a pair of duplicated genes (or paralogous genes). Converted tracts will be pasted to the corresponding part of the paralog, while there is no change in the original gene. Conversion can result in the novel mutation (shown in red) being spread or reversed. Although converted tracts will become identical in sequence, conversion will create novel haplotypes that are chimeras of the two genes (shown in purple and yellow).

The outcome of a single gene conversion event between a pair of paralogous sequences is that the fragment of the sequences that was subject to the conversion event (conversion tract) becomes identical. This means that if either paralog has accumulated a mutation e.g., a substitution or small indel), this mutation will either be transferred and thus shared between the paralogs or reversed, depending on the direction of the conversion event (Figure 1). The occurrence of gene conversion requires the pairing between two highly similar sequences of sufficient length (typically a few hundred nucleotides). The conversion rate is generally higher when the distance between the paralogs is smaller, although conversions do occur between interchromosomal paralogs, albeit less frequently [16]. The length of the conversion tract can range from a few nucleotides to several hundred nucleotides or more [18] (see also Mansai *et al.* in this issue), and gene conversion can occur recurrently in varying tracts across the whole duplicated region.

### 2.2. Divergence

**Figure 2:**
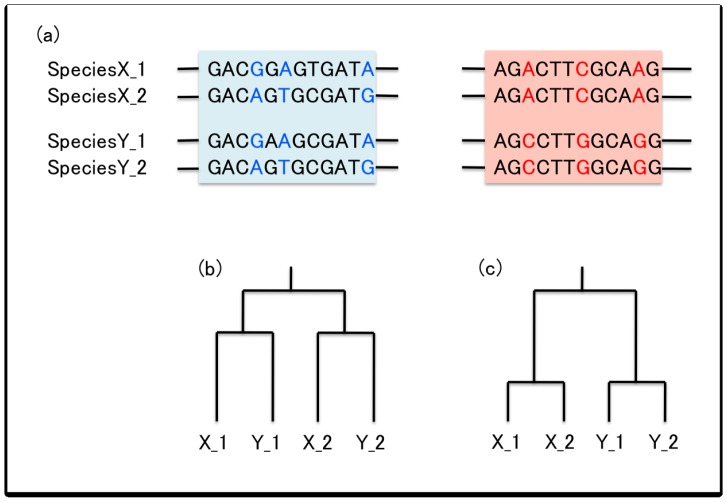
Illustration of the sequence evolution after gene duplication. (**a**) Paralogs in species X and Y that originated by a duplication before the divergence of X and Y. The region in blue and red are evolving without and with gene conversion, respectively. (**b**) Gene tree without gene conversion, which reflects the true evolutionary history. (**c**) Gene tree with frequent gene conversion.

Gene conversion can result in various outcomes that differ from what is expected under evolutionary models based on the classical concept of molecular clock which assumes that the different genes evolve independently. One well-known outcome is concerted evolution, in which the divergence between the duplicated genes undergoing frequent gene conversion will remain low. By contrast, the divergence will be expected to increase roughly in a linear fashion if the genes accumulate substitutions independently. To further explain this difference in the pattern of divergence with and without gene conversion, let us consider a duplication event that occurred in the common ancestor of species X and Y where the duplicates X1, X2, and Y1, Y2 are retained in both species. Thus, X1 and Y1 are orthologous, and likewise with X2 and Y2 (Figure 2). If the duplicates have been evolving independently, the divergence between whichever paralogous gene pair would correspond to the time since the duplication event. In such cases, a number of substitutions shared by the orthologous genes, *i.e.*, those that occurred before the divergence of species X and Y should be observed (nucleotides in blue in Figure 2a). However, with gene conversion which homogenizes the paralogous sequences, a number of substitutions shared by the paralogous genes X1 and X2, or Y1 and Y2 should be observed (nucleotides in red in Figure 2a). The divergence between the duplicated genes would be much lower than expected under the assumption of a molecular clock and thus the duplication event would appear to be much “younger” than it actually is, assuming a linear increase of the number of substitutions over time.

**Figure 3:**
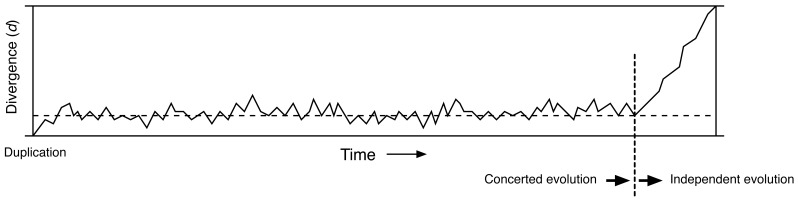
Typical behavior of the paralogous divergence *d* since the duplication event. *d* first increases to an equilibrium value represented by the broken line, then drifts around it. During this phase, the duplicates are considered to be undergoing concerted evolution. When concerted evolution is terminated, *d* starts increasing roughly linearly.

Gene conversion occurs between duplicated sequences until the pairing is disrupted due to the accumulation of multiple substitutions or the insertion of transposable elements (TEs) and other large indels. This is one of the major factors which determines the trajectory of molecular divergence between paralogs. Suppose that the divergence between a pair of duplicates (*d*) is the proportion of sites in the nucleotide sequence that differs between the two sequences, which is obviously zero when the duplication occurred. A typical behavior of *d* is illustrated in Figure 3. *d* first increases, and in a relatively short time enters a phase where it fluctuates because the two opposing forces are in balance. One is the mutational pressure that increases divergence and the other is gene conversion that decreases divergence. During this phase, the duplicates are considered to be undergoing concerted evolution. The expected value of *d* in this phase of concerted evolution is theoretically given by a function that mainly involves the mutation rate and gene conversion rate [19,20]. The gene conversion rate will change depending on the divergence between the paralogous sequences and other factors mentioned above, and the phase of concerted evolution will be terminated when gene conversion is somehow restricted. Once concerted evolution is terminated, the duplicates can accumulate mutations independently so that *d* increases over time (*i.e.*, *d* for neutral mutations follows a molecular clock) [21]. It should be noted that along the process in Figure 3, the sequences of the paralogs continuously diverge from the original sequence at the time of duplication. This is why we observe divergence between orthologs from different species (Figure 2). Under neutrality, the orthologous divergence follows a molecular clock, and theoretically the rate of neutral nucleotide substitution (evolutionary rate) is identical to that of single-copy genes (see below for the cases with selection).

### 2.3. Polymorphism

**Figure 4:**
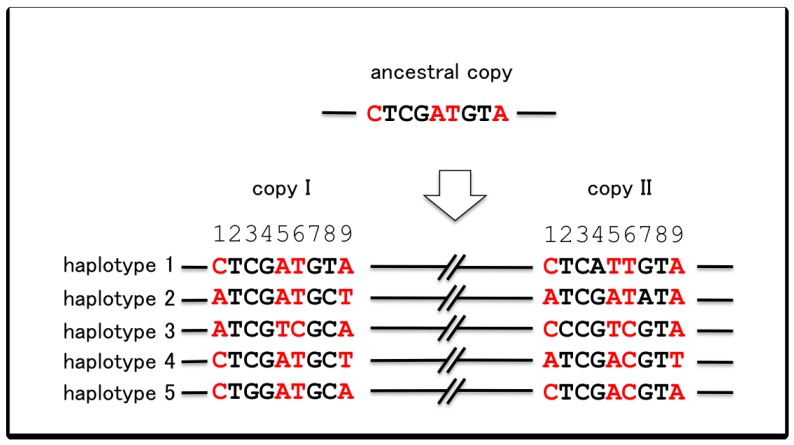
Hypothetical polymorphism data from a pair of duplicated genes with sample size 5. Positions where the nucleotides are in red contain individuals that have the same (shared) substitutions in both copies, referred to as shared polymorphisms. For instance, in position 1, haplotype 2 contains the substitution of C to A in both copies. The same applies to haplotype 3 in positions 5 and 6 (AT to TC), and haplotype 4 in position 9 (A to T).

Another outcome of gene conversion is manifested as a pattern of polymorphism referred to as “shared polymorphism” (Figure 4). Suppose that one of the duplicated gene accumulates a mutation in a particular lineage. This mutation will increase or decrease in frequency within the population until it becomes fixed or eliminated. If there is no gene conversion, such a mutation will only be observed as a polymorphism in the copy in which it occurred. However, if gene conversion occurs, this mutation will be transferred to the paralogous copy and the same mutation will be observed as a polymorphism in both copies (shown in red in Figure 4). This is called a shared polymorphism, which is not expected to occur without gene conversion as the chance for the same mutation to occur independently in both copies should be extremely low. Shared polymorphism is a very strong indicator of gene conversion and has been used to infer gene conversion in many studies [20,22,23].

Although duplicated genes undergoing gene conversion will remain highly similar to each other, gene conversion can create several new haplotypes. Consider the example in Figure 1. The gene in purple has acquired two new tracts from the gene in yellow, resulting in a new haplotype containing fragments from the gene in yellow, one of which has a novel mutation T in the position of G, thus creating a chimera of the two genes. One can imagine that frequent gene conversion of different tracts will create several new haplotypes that would not be possible without gene conversion. This may be especially enhanced in larger gene families.

## 3. Non-Neutral Evolution of Duplicates with Gene Conversion

So far, we have described the outcome of interlocus gene conversion and its effect on the evolution of duplicated genes without considering selection on gene conversion. In this section, we focus on how selection works for and against gene conversion. As with any other mutational process, gene conversion can have positive or negative effects, which largely depends on what kind of mutation is involved in the conversion tract. In other words, in most cases selection targets the mutation rather than the conversion event itself. There are many possible situations where gene conversion is selectively preferred or disfavored, but most cases can be theoretically handled by two simple forms of selection. To explain the evolutionary dynamics with selection in the framework of population genetics, we here consider a simple model where all individuals in a diploid population with size *N* have a pair of duplicated genes. Two different alleles, the ancestral variant A and new derived allele B, are allowed. Consider the initial state in which haplotype AA is fixed in the population, where the two characters represent the alleles at the first and second copies of the duplicate pair. We assume that the new variant B has a slightly different function from A (either B improves or degenerates the original function or B has a novel function). When B arises in one individual in the population, a new combination of AB is created (no distinction will be made between AB and BA). Gene conversion homogenizes the allelic variation between the two copies, such that it works to spread the derived state B to the other copy, thus creating BB, or to reverse the derived state B to its original state A, thereby creating AA. In this simple framework, we consider how selection works for and against gene conversion under different conditions together with the effect of gene conversion in the molecular evolution of duplicated genes. As is described below in detail, the two major modes of selection are: (i) Directional selection for or against the new variant B; (ii) Selection to maintain genetic variation so that AB and BA are preferred to AA and BB.

### 3.1. Directional Selection

Whether gene conversion has a positive or negative effect largely depends on the nature of the derived state B, *i.e.*, the effect of the new mutation, and the direction of the conversion (AB to BB or AB to AA). For instance, if the new mutation is disadvantageous such as by disrupting the gene function, a conversion that restores the original state AA would be beneficial. Katju *et al.* [24] reported one such example where a loss-of-function mutation in the *fog-2* gene of *Caenorhabditis elegans*, which results in a change in reproductive system [25], is frequently repaired by gene conversion between fog-2 and its neighboring paralog *ftr-1*. Conversely, conversion will have a negative effect if such a new disruptive mutation is transferred to another functional copy. Loss-of-function mutations introduced by gene conversion with a pseudogene have been implicated in various human genetic diseases (see [18] and [26]). On the other hand, when B has an improved function, selection should work to spread B, thereby increasing the fixation probability of BB.

The potential benefit of gene conversion in spreading an advantageous mutation or eliminating a deleterious mutation can be especially enhanced when retention of the sequence similarity between the duplicates is favored. In such cases, we can say that AA or BB is favored over AB, and gene conversion in whichever direction is likely to be beneficial. Selection should work through gene conversion to maintain AA when B is not beneficial and to create BB when B is beneficial. Theoretically speaking, the model would be similar to Kimura's compensatory mutation model [27]. One important evolutionary advantage of gene duplication is increased gene dosage, and the retention of sequence similarity is likely to be beneficial when there is selection to produce more of the same protein. Sugino and Innan [28] noticed that there was a large variation in the duration of concerted evolution among duplicated genes in *Saccharomyces cerevisiae* that were created by the whole-genome duplication (WGD) [29,30]. A positive correlation between the duration of concerted evolution and the gene expression level was found, but not with other factors such as amino acid substitution rates, and they proposed that selection works to maintain the process of gene conversion in duplicated genes in which higher dosage is favored. The human Y chromosome contains many genes that undergo frequent gene conversion [8]. These genes are expressed in the testis and are thought to be under strong selective pressure for higher dosage because of sperm competition [31]. The ribosomal genes and histone genes also require high dosage and appear to undergo frequent gene conversion [32,33].

The recent study of Evangelisti and Conant [34] shed some new light on the role of gene conversion in the ribosomal duplicates in yeast that were created by the WGD. First, they showed that most WGD-derived ribosomal gene duplicates are indeed subject to gene conversion by comparing their non-synonymous divergence with the divergence between orthologs of an outgroup species. However, little evidence of gene conversion was found in the noncoding regulatory regions and the expression pattern of most of the duplicates were found to have diverged. This is puzzling because if the similarity were retained because of selection for increased dosage, one would imagine that the expression pattern would overlap in order to actually achieve higher dosage. The authors also noted previous studies showing that the ribosomal gene duplicates are not functionally interchangeable despite their very high protein sequence identity [35]. In order to explain these observations, they proposed the following hypothesis. According to their hypothesis, the ribosomal duplicates have subfunctionalized at the expression level due to mutations in the regulatory non-coding regions. Nevertheless, there is still very strong purifying selection to preserve the ancestral function of the ribosomal genes so that the proteins encoded by the different paralogs can substitute each other under the different expression conditions. This would require high protein similarity and therefore gene conversion would be favored.

One can imagine that gene conversion might provide an additional platform on which selection can exert its influence on the fate of novel mutations in a way that would not be possible if different copies were evolving independently without gene conversion. For instance, gene conversion might allow a more efficient removal of disruptive mutations and spread of beneficial mutations in multigene families. The recent work of Mano and Innan [36] provided a theoretical framework for this idea. They studied the fixation process of a mutation that spreads in a multigene family by gene conversion and the effect of selection, and demonstrated that gene conversion serves to increase the effective population size, thereby enabling more efficient selection. Their theory predicts that in the presence of gene conversion, sites in multigene families under positive selection would evolve faster in comparison with a single copy gene, whereas sites under purifying selection would evolve slower. Note that the evolutionary rate at neutral sites is identical for duplicates and single-copy genes. Recent studies have explored this role of gene conversion in enabling more efficient selection using the human Y chromosome as a model [37,38]. Selection is predicted to be inefficient on the non-recombining Y chromosome, and as a result, the Y chromosome is thought to be undergoing degeneration [39]. However, many genes on the Y chromosome belong to large gene families and the level of gene conversion is extremely high within these gene families. This might enable the removal of deleterious mutations and the spread of beneficial mutations in the absence of recombination, thereby preventing the degeneration of Y-linked genes [8,31]. Based on simulations, it was indeed shown that high rate of gene conversion is beneficial for the Y chromosome because it allows more efficient removal of deleterious mutations, which helps to prevent the degeneration [37,38].

### 3.2. Selection to Maintain Genetic Variation

There could be some cases where haplotypes having both alleles (*i.e.*, AB and BA) might be favored over AA or BB, even if B itself might be beneficial. This might occur if B encodes for a novel function and it is beneficial to retain B but also the ancestral A, or in gene families that favor diversification so that selection will work to maintain a novel function that arises in one copy. In such cases, gene conversion itself will be disadvantageous as it serves to homogenize the duplicates, regardless of the direction of the conversion. Note that this differs slightly from cases where gene conversion in one direction is advantageous (AB to BB; sharing beneficial mutations) but disadvantageous if it occurs in the other direction (AB to AA; reversing beneficial mutations). Duplication and rearrangements that result in duplicated genes being located on different genomic locations (e.g., different chromosomes) can be one way that allows genes to escape homogenization and acquire divergence [40], as in many species gene conversion is less likely to occur between copies on different chromosomes [11,13,41]. This has been suggested to have contributed to the diversification of the NBS-LRR genes, a large plant resistance gene family, in *Arabidopsis* [42].

**Figure 5:**
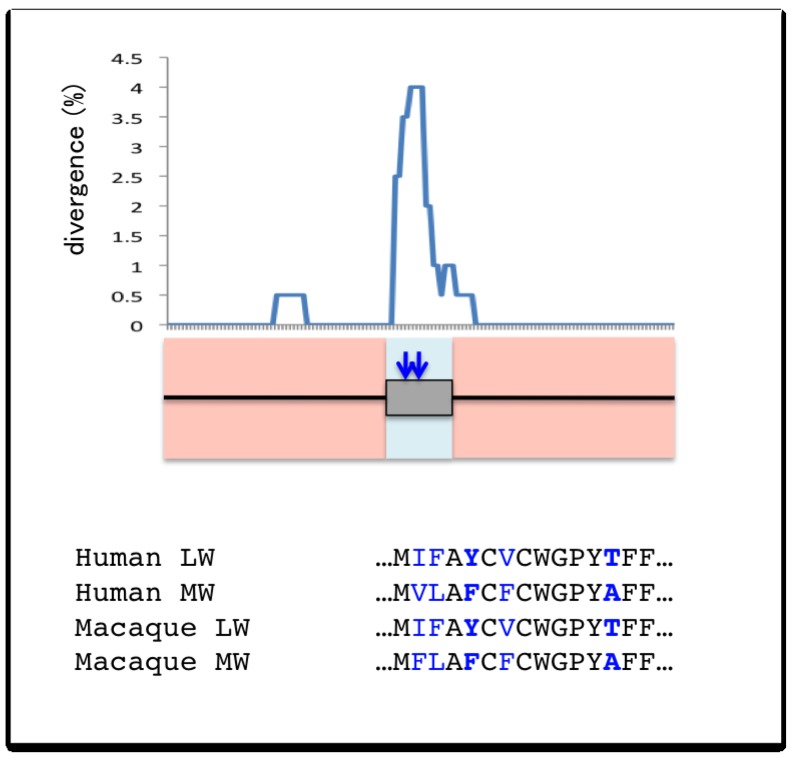
Sequence divergence of the red- and green-opsin genes in human and macaque. The graph shows the divergence between the human red-opsin (long-wave: LW) and green-opsin (medium-wave: MW) genes of intron 4, exon 5, and intron 6. The divergence data was taken from [46]. The gene structure of the region is shown below with the introns 4 and 6 represented by black lines and the exon 5 represented by a gray box. Regions where the similarity is higher between paralogs of the same species than between orthologs of other species (*i.e.*, the introns) due to frequent gene conversion is in a light red background, whereas regions where the similarity is higher between the orthologs than the paralogs (*i.e.*, the exon) are in a light blue background. The two blue arrows indicate the positions of the two fixed amino acid substitutions that are largely responsible for the difference in color sensitivity [53]. The amino acid sequences of the red-opsin (LW) and green-opsin (MW) genes of human and macaque [49] around the two functionally significant substitutions are shown below. The sites in blue are sites where the difference is fixed between either of the orthologous pair, and the sites of the two functionally significant substitutions are in bold.

Innan [43] showed that even if the two copies are located closely and subject to frequent gene conversion, the two alleles can be maintained in the genome for a long period of time and divergence can accumulate if selection is sufficiently strong. If the target site of selection, *i.e.*, the region where divergence is favored is small, this can result in the divergence between the duplicated genes being high in certain regions of the gene but very low in other regions [43–45]. It was recently shown that a peak of divergence can be a useful signature for detecting such target sites of selection in duplicated genes that are undergoing gene conversion [46]. This was demonstrated in human red- and green-opsin genes. There is good evidence suggesting that these genes residing ∼24 kb apart from each other were duplicated ∼30–40 mya (million years ago) and that high sequence similarity is maintained by ongoing frequent gene conversion [47–50]. Gene conversion is particularly prevalent in introns, whereas a number of differences have been fixed in some exons. As a result, when the coding sequences are compared, the sequences of different species such as human and macaque are more similar to each other than to the paralogs in the same species (Figure 5). By contrast, when the intronic sequences are compared, the duplicates in the same species are more similar to each other than to their orthologs in other old-world monkeys [49,51,52]. Of particular significance are two amino acid substitutions located close to each other on exon 5, which are largely responsible for the difference in color sensitivity [53]. Interestingly, the divergence increases in exon 5, while being maintained at low levels in the flanking introns, creating a large peak of divergence around these two sites of functionally important changes (Figure 5) [46]. This implies that strong selection restricted gene conversion in this region in order to preserve the newly arisen function. This idea was also used to determine sites where differences are maintained by selection under the pressure of gene conversion in duplicated genes in *Drosophila* and yeast [12,54].

There is another form of selection on gene conversion to maintain genetic variation, although the situation is so complicated that it cannot be described in a simple two-allele model. In some gene families, selection favors a large amount of genetic variation at the haplotype level, and not necessarily the sequence diversity. In such gene families, gene conversion can work to increase the haplotype variation by introducing novel haplotypes. This important role of gene conversion has been demonstrated in several gene families such as the major histocompatibility complex (MHC) genes, plant self-incompatibility (SI) genes, and immunoglobulin genes. These genes typically have many paralogs, and gene conversion is highly active between them [55–61] (see also [45] in this issue). Takuno and Innan [62] recently showed that in such gene families in which haplotype diversity is highly favored, nonfunctional copies can be preserved in the genome if gene conversion is active, as they can serve as templates to create novel haplotypes through the action of gene conversion. This result is consistent with the fact that there are a number of pseudogenized duplicated genes in the MHC cluster where there are a number of clear footprints of gene conversion between pseudogenes and their functional donors. Other examples include plants' disease-resistance genes (*R* genes). Similar to the MHC genes, the *R* multigenes usually form a cluster in which many pseudogenes exist, and gene conversion among them has been implicated [63]. Furthermore, this beneficial effect of gene conversion in the creation of haplotype variation can also hold in somatic cell divisions in the immunoglobulin genes [55,64,65]. It is known that somatic gene conversion from pseudogenized duplicates to the functional copy contributes substantially to the high levels of variation in the antigen-binding domain [66–69] (see also [70] in this issue).

## 4. Interpreting Molecular Data

When gene conversion influences the molecular evolution of duplicates as described in the previous sections, the misinterpretation of data is a common occurrence. The major problem in such misinterpretation is that the effect of gene conversion is not seriously considered and standard analyses based on a molecular clock are automatically applied to the divergence between duplicates. Here, we use some examples to demonstrate the importance of analyzing data with gene conversion taken into account.

One of the most extreme example is the short arms' termini of rice chromosomes 11 and 12, which were duplicated by a WGD event that occurred in the common ancestor of most grass species. The entire region of ∼3 Mb (including the intergenic regions) maintains a very high sequence similarity, despite being as old as ∼70 million years ago (mya) [71]. This region was initially thought to have originated by a segmental duplication <10 mya [72,73], but was recently found to be shared by several other grass species such as sorghum [71]. Interestingly, the orthologous regions in other grass species which share the WGD also appear to have undergone more extensive gene conversion compared to the rest of the genome, although to a varying degree depending on the species [71,74,75]. One notable feature of this region is that it harbours very few TEs [74], although further investigation is required to better understand the evolutionary dynamics of this region. Thus, this example shows that a huge region including non-coding regions can undergo long-term concerted evolution.

The situation is quite different in the case of yeast species. The baker's yeast *S. cerevisiae* has experienced a WGD roughly 100–200 million years ago [29,30]. There are a number of duplicates that have undergone concerted evolution since the WGD, but for almost all the cases the region under concerted evolution is restricted to the coding region, and it is extremely difficult to detect homology between the paralogs in non-coding regions (Sugino and Innan, personal communication). It seems that gene conversion was silenced in non-coding regions a long time ago, resulting in a striking difference in the pattern of molecular evolution between coding and non-coding regions. The most likely cause of this difference is the intensity of purifying selection, which is much stronger in coding regions so that it maintains the sequence similarity, which likely increases the efficiency of gene conversion.

**Figure 6:**
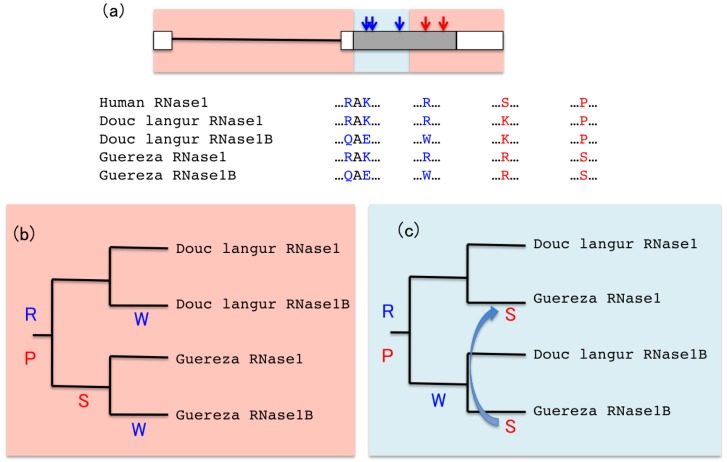
Different possible scenarios of the evolution of the RNase genes in colobine monkeys. Douc langur (Asian colobine) and Guereza (African colobine) both contain duplicates of the RNase1 gene, designated here as RNase1B. (**a**) Examples of sites containing amino acid substitutions that are shared across species but not within species, and those that are shared within species but not across species are shown in blue and red, respectively. The sequences are taken from [76]. Although [76] shows two duplicated genes in Guereza, designated as 1*β*and 1*γ*, the sites shown here are all sites that are identical in both 1*β* and 1*γ*. The exon-intron structure is shown above with the UTRs in open boxes and the coding region in a gray box. The positions of the sites containing substitutions shared across species but not within species are indicated by blue arrows, and those shared within species but not across species are indicated by red arrows. Regions where the similarity is higher between sequences of the same species than between sequences of other species is in a light red background, whereas regions where the similarity is higher between the sequences across species than sequences within species are in a light blue background. (**b**) One possible scenario—that the duplication occurred after the divergence of the two species. (**c**) Another possible scenario—that the duplication occurred prior to the divergence of the two species. One example each of amino acids shared across species but not within species (R and W in blue), and shared within species but not across species (P and S in red) are shown. The ancestral state is shown at the root of the tree and the branches in which the substitutions would have occurred according to each scenario are indicated. The arrow indicates gene conversion, although the direction is hypothetical and could have occurred in either direction.

Another intriguing example is the evolutionary history of the RNase gene duplicates in the colobine monkeys where the sequence data has been interpreted in constrasting ways depending on whether independent evolution or frequent gene conversion is assumed [76–79]. The colobine monkeys are leaf-eating and adopt a ruminant-like digestive system, which requires different enzymatic activities compared to most other primates which typically eat fruits and insects [80]. The Asian colobine, douc langur (*Pygathrix nemaeus*), and the African colobine, guereza (*Colobus guereza*), both have two (or more) copies of RNase genes [76,77]. As illustrated in Figure 6, there are three identical amino acid substitutions in the duplicates of both the Asian and African colobine monkeys. The amino acids at the three sites in the original copy (RNase1) are identical to those in humans, indicating that the three amino acid substitutions were accumulated in the lineage of the derived copy. Zhang [76] elegantly demonstrated that the enzyme activity difference between the duplicates can be largely explained by the three amino acids, and suggested that these amino acid fixations occurred most likely by positive selection related to their leaf-eating lifestyle.

Zhang [76] also performed evolutionary analyses of these genes. He constructed phylogenetic trees with the entire region containing both coding and non-coding sequences of these duplicates of the different species, which showed that the two duplicates in each species are more closely related to each other than to the duplicates in the other species. This topology was interpreted such that the duplicates in each species arose independently after the divergence of the Asian and African colobines and that the two duplicates had independently acquired the three amino acid substitutions in each clade. He suggested that these substitutions were parallel substitutions driven by the same strong selective pressure, and this study drew a great deal of interest because parallel evolution at the molecular level was considered to be rare [81]. Subsequently, Yu *et al.* conducted similar phylogenetic analyses using many other colobine monkey species and identified four additional substitutions that were shared between duplicates across different species, and suggested that these were all parallel amino acid substitutions that occurred independently in different colobine monkeys [79].

However, an alternative evolutionary scenario that can also explain these hypotheses of parallel amino acid substitutions has been proposed. This alternative hypothesis posits that one duplication event occurred before the divergence of the two clades, and that the duplicates of the same species cluster together because their sequences are homogenized by gene conversion [77,78]. Schienman *et al.* who analyzed a larger number of species suggested that this is more parsimonious than having to infer a large number of independent duplications and deletions in different species (note that some species do contain other additional lineage-specific duplications) [77]. Under this scenario, the identical amino acid substitutions in various species can simply be explained as substitutions that occurred in the duplicates before the divergence of the different species. If such novel mutations of high adaptive significance arise, as experimentally verified by Zhang [76], it is plausible that selection would maintain such mutations by restricting gene conversion in the region surrounding these substitutions as we discussed in the previous section. Interestingly, the divergence between the duplicates is much lower in the non-coding sequences (intron and UTRs) than in the coding sequences [77,78]. In fact, phylogenetic trees constructed using only the coding region result in trees with much lower bootstrap support [79], or different topologies where duplicates of the same species do not cluster together [77,78]. This is reminiscent of the example of the opsin genes discussed above (Figure 5), where strong selection to retain the adaptive mutations has resulted in the divergence restricted to the coding region, despite the divergence being low due to gene conversion in the introns [46]. Although Zhang [82] and Yu *et al.* [79] both argued that trees constructed by combining the coding and non-coding regions should be more accurate because a larger number of nucleotides are used, the issue is not the ‘accuracy’ (or high bootstrap value) of the tree, which can only be as good as the sequence information that is provided. If divergence accumulates in a relatively narrow region, such information will likely be masked if the entire region is treated together as if it shares the same evolutionary history. The interpretation regarding the occurrence of gene conversion between these genes also differs between the different studies. Schienman *et al.* found that a 5 bp deletion in the coding region of one of the copies was also present in an allele of the other copy in the African colobine [77], which is usually considered a signature of gene conversion. On the other hand, Zhang [82] and Yu *et al.* [79] could not detect any gene conversion with GENECONV [83]. Clearly, further investigation is required to resolve these different scenarios. Zhang's hypothesis [76] can be verified if the duplicates are located in different genomic locations in the two different clades, which will make it highly likely that they are independent duplications. On the other hand, if the duplicates are located in the same genomic locations with conserved synteny in the different species, it will be most likely that they originated by a single duplication in the common ancestor. Unfortunately this has not been possible with these genes due to the lack of genomic data [78].

As we have discussed, low sequence divergence caused by gene conversion results in a younger estimate of the duplication date, which can in turn lead to overestimation of the number of duplication events when placed in a phylogenetic context. However, such misinterpretation can be avoided in some cases by examining the genomic collinearity of multiple species. In the case of the duplicated rice region, the identification of duplicated, collinear regions in sorghum [71] and other species [75] served to clarify that the duplication event was shared with several other grass species and much more ancient than assumed based on sequence divergence. Gao and Innan were also able to show that several yeast duplicates that appear young based on the divergence of synonymous sites were actually much older by identifying both copies in orthologous regions of other yeast species [9]. The main reason why the evolutionary scenario of the RNase genes has not been resolved is because a reliable assignment of orthology and paralogy based on genomic data has not been possible. The conclusions of Zhang [76] and Yu *et al.* [79] depend on phylogenetic methods, which are prone to the influence of gene conversion. On the other hand, many statistical tests to detect gene conversion require prior knowledge regarding the ‘true’ evolutionary relationships between the genes in question. Without such information, our interpretation ultimately comes down to how frequently we expect gene conversion or parallel mutations to occur. A careful assignment of orthology and paralogy, that means not solely based on any sequence similarity methods that will be highly influenced by gene conversion or in some cases by parallel substitutions, but based on genomic collinearity and perhaps also by examining flanking sequences should allow us to infer the molecular evolution of duplicates in a much more reliable way. Another important point in order to avoid misinterpreting the data is the accuracy in detecting gene conversion. Zhang [82] and Yu *et al.* [79] both did not detect any conversion between the RNase genes using GENECONV and thus concluded that there was no gene conversion and that the duplicates were created independently in both lineages. Although the methods for detecting gene conversion are discussed elsewhere [84] (and Mansai *et al.* in this issue), it is worth noting that despite the usage of GENECONV being common practice, it is known to have a high false-negative rate when gene conversion is frequent and covers a large portion of the duplicates, which might be the case with the non-coding region of these RNase genes. As always is the case, absence of evidence is not evidence of absence; therefore, it is important to consider the occurrence of gene conversion using various approaches.

## 5. Conclusions

In this article, we have described how gene conversion allows the evolution of DNA sequences of duplicated genes in a way that is not possible by independent evolution, which can be observed in the patterns of sequence divergence and polymorphism. As we illustrated using the examples of the rice duplicated region and the RNase genes in colobine monkeys, ignoring the possible effect of gene conversion can potentially result in misinterpreting molecular data, especially because regions subject to gene conversion will appear much younger than based on a molecular clock assumption. Although this underestimation of the age of duplication is probably the best known “problem” caused by gene conversion, it was recently shown that the divergence pattern created by gene conversion can be misinterpreted as positive selection [85]. GC-biased gene conversion is another potential factor to create false positive evidence for positive selection [86,87]. These effects should also be kept in mind in future studies of duplicated genes. We also discussed how selection works on duplicated genes in the presence of gene conversion. It appears that gene conversion might work to enhance the evolutionary potential of duplicated genes by providing an additional substrate that natural selection can make use of. Many recent studies have attempted to estimate the genome-wide occurrence of gene conversion (e.g., how many percent of all genes have undergone gene conversion) [9,11–13,88–90] (see also other articles in this issue). However, the estimates vary considerably between various studies, and the extent of the contribution of gene conversion is still unclear. This is largely due to the difficulty in detecting gene conversion, as those that GENECONV or phylogeny-based methods can detect might be rather restricted (see [84]), and ancient gene conversion events will be rather difficult to detect. In addition, multigene families are often difficult to include in detailed analyses or to incorporate into theoretical frameworks [14], which also might add to the discrepancies between different studies. Thus, additional challenges lie ahead in estimating the extent of gene conversion in various species. Furthermore, it will be of great interest to better understand the contribution of selection to the pattern of evolution under the pressure of gene conversion.
